# Primary biliary cholangitis with features of autoimmune hepatitis in a 19-year-old adolescent with 14q24.1q24.2 deletion: a case report

**DOI:** 10.3389/fped.2023.1280409

**Published:** 2023-12-12

**Authors:** Yi-Wei Wang, Hsun-Chin Chao, Huei-Shyong Wang, Ju-Li Lin, Chih-Chen Chang, Shiu-Feng Huang

**Affiliations:** ^1^ Division of Pediatric Gastroenterology, Department of Pediatrics, Chang Gung Children’s Medical Center, Chang Gung Memorial Hospital, Taoyuan, Taiwan; ^2^ Division of Pediatric Neurology, Department of Pediatrics, Chang Gung Children’s Medical Center, Chang Gung Memorial Hospital, Taoyuan, Taiwan; ^3^ Division of Endocrine & Medical Genetics, Department of Pediatrics, Chang Gung Children’s Medical Center, Chang Gung Memorial Hospital, Taoyuan, Taiwan; ^4^ Department of Medical Imaging and Intervention, Chang Gung Memorial Hospital, Taoyuan, Taiwan; ^5^ Division of Molecular and Genomic Medicine, Department of Pathology, National Health Research Institute, Miaoli, Taiwan

**Keywords:** case report, adolescent, primary biliary cholangitis, autoimmune hepatitis, autoantibodies, 14q interstitial deletion, array CGH

## Abstract

**Introduction:**

Primary biliary cholangitis (PBC) is a rare and chronic autoimmune liver disease characterized by the progressive destruction of small intrahepatic bile ducts that may eventually lead to cirrhosis. PBC with features of autoimmune hepatitis (AIH) has rarely been reported in pediatric patients with genetic defects. We present the case of an adolescent with chromosome 14q24.1q24.2 deletion who was given the diagnosis of stage IV PBC with features of AIH.

**Case presentation:**

A 19-year-old male adolescent with multiple congenital abnormalities and an intellectual disability presented with abnormal liver enzymes levels and pruritus for more than 5 years. Laboratory examinations revealed elevated levels of aspartate aminotransferase, alanine aminotransferase, alkaline phosphatase, and gamma-glutamyl transpeptidase. After the exclusion of viral hepatitis, alpha-1 antitrypsin deficiency, Wilson's disease, and other genetic cholestatic liver diseases by laboratory tests and whole exome sequencing, a liver biopsy was performed and stage IV PBC was diagnosed. Notably, features of AIH were also noted in the histopathological report, indicating the presence of PBC with AIH features. The patient responded well to a combination therapy of ursodeoxycholic acid and steroids. Array comparative genomic hybridization analysis performed to study the congenital abnormalities revealed a 3.89 Mb 14q24.1q24.2 deletion.

**Conclusion:**

PBC with AIH features has rarely been reported in an adolescent with a chromosomal abnormality. The present case can increase awareness for early-onset PBC and its possible correlation with chromosomal defects.

## Introduction

Primary biliary cholangitis (PBC), formerly known as primary biliary cirrhosis, is a rare and chronic autoimmune liver disease that predominantly affects middle-aged women. It is characterized by the progressive destruction of small intrahepatic bile ducts that may eventually lead to cirrhosis and liver failure (1). Although patients with PBC commonly presents with fatigue and pruritus, up to 50%–60% of patients have asymptomatic anicteric cholestasis (2). Antimitochondrial antibodies (AMAs), particularly the M2 subtype (AMA-M2), are the serological hallmark of PBC and are rarely associated with other clinical conditions. AMAs are present in approximately 95% of patients with PBC; however, some patients may have AMA-negative PBC (3). Although the etiology of PBC remains elusive, the interplay between environmental and immunological factors in an individual with genetic susceptibility is thought to lead to the characteristic pathological and clinical phenotypes of the disease. In a meta-analysis, the pooled global incidence and prevalence of PBC were reported to be 1.76 and 14.60 per 100,000 people, respectively (4). The coexistence of autoimmune hepatitis (AIH) with PBC in the same patient is referred to as PBC with AIH features or PBC–AIH overlap syndrome; it accounts for approximately 5%–15% of all PBC cases (1). Unlike other autoimmune liver diseases, such as AIH or primary sclerosing cholangitis, both PBC and PBC with AIH features have rarely been reported in pediatric patients. To the best of our knowledge, few case reports are available in the literature (5–13). Among the reported pediatric cases, only one case had chromosomal abnormalities (8), and only one had coexisting AIH (9). Herein, we report the case of an adolescent with 14q24.1q24.2 deletion who was given the diagnosis of PBC with AIH features.

## Case report

The patient was a 19-year-old male who was born late preterm at 36 + 5 weeks of gestation to healthy non-consanguineous Taiwanese parents. His birth weight was 3,410 grams, and his Apgar score was 8 at 1 min and 9 at 5 min. Multiple congenital abnormalities, including left thumb polydactyly, webbed neck, and facial dysmorphism, were detected at birth. A series of ultrasound examinations showed 5-mm patent ductus arteriosus (PDA) and left ectopic kidney located at the left side of the pelvis. The patient underwent surgical ligation for hemodynamic significant PDA smoothly without complications.

The patient had neurodevelopmental delay with intellectual disability and autism spectrum disorder. Several examinations were performed during his early childhood to determine the possible underlying causes. Chromosome analysis revealed a 46, XY karyotype. The results of tandem mass screening for metabolic disease and fragile X syndrome were normal. The patient had a healthy older sister and a family history of thalassemia. No chromosomal abnormality, neurodevelopmental disorder, autoimmune disease, or liver disease were noted among his family members. Throughout his childhood, especially before 10 years of age, the patient was admitted multiple times to our hospital, mostly because of bronchopulmonary infection. Laboratory examinations during these hospital stays revealed mild increases in his aspartate aminotransferase (AST) and alkaline phosphatase (ALP) levels, which first became apparent when the patient reached the age of 3 years (Table 1). Therefore, ceruloplasmin levels were assessed and found to be within the normal range at 30.8 mg/dl (normal range: 18–45 mg/dl). As he grew older, he was less frequently hospitalized; however, after he turned 13 years old, he began to experience unexplained pruritus. Over the past two years, mild postural tremor and ataxia were also observed.

**Table 1:** Laboratory results of the patient since childhood, during hospital stay in 2022, and after treatment.

	Reference range	Laboratory results in different ages (yr)							Days of hospitalization (days)					Months after treatment^c^ (mo)			
		6 mo	3	5	7	9	16	18	−7	1	10^a^	20^b^	30	1	4	6	12
AST (U/L)	<2 yr: 9–80	79	**65**	**56**	**54**	**62**	**109**	**119**	**105**	**90**	**61**	**76**	**141**	**55**	**57**	**70**	**64**
	≥2 yr: ≤34																
ALT (U/L)	≤36	NT	31	19	29	24	**53**	**81**	**66**	**43**	29	36	**52**	**50**	**48**	**51**	**54**
ALP (U/L)	<12 yr: 116–515	NT	**576**	NT	**609**	NT	**525**	196	**237**	**234**	**201**	**223**	**259**	**216**	**140**	**190**	**241**
	16–19 yr: 58–237																
	≥19 yr: 28–94																
GGT (U/L)	<71	NT	NT	NT	NT	NT	NT	NT	**670**	**630**	**517**	**388**	**476**	**415**	**417**	**548**	**467**
T. Bil (mg/dl)	<1.2	NT	0.9	NT	**1.6**	NT	NT	NT	1.1	**1.2**	**1.4**	**1.3**	**1.4**	1.1	0.6	0.8	0.8
D. Bil (mg/dl)	<0.4	NT	0.3	NT	NT	NT	NT	NT	**0.5**	**0.5**	**0.5**	**0.7**	**0.5**	**0.5**	0.3	0.3	0.3
Cu (µg/dl)	70–140	NT	NT	NT	NT	NT	NT	NT	NT	NT	**159**	NT	NT	104.6	NT	**146.2**	132.8
Mn (µg/L)	<2.4	NT	NT	NT	NT	NT	NT	NT	NT	NT	**2.6**	NT	NT	1.8	NT	2.2	2.2
AMA-M2	Neg	NT	NT	NT	NT	NT	NT	NT	NT	NT	Neg	NT	NT	NT	**Pos**	Neg	Neg
Other AAbs	Neg	NT	NT	NT	NT	NT	NT	NT	NT	NT	Neg	NT	NT	NT	**SLA/LP Equ**	Neg	Neg

The results in bold are abnormal.

Yr, years; Mo, months; AST, aspartate aminotransferase; ALT, alanine aminotransferase; ALP, alkaline phosphatase; GGT, gamma-glutamyl transpeptidase; T. Bil, total bilirubin; D. Bil, direct bilirubin; Cu, copper; Mn, manganese; AMA-M2, antimitochondrial antibody M2 subtype; AAbs, autoantibodies; NT, not tested; anti-SLA/LP, anti-soluble liver antigen/liver–pancreas antibodies; Neg, negative; Pos, positive; Equ, equivocal.

^a^ During hospitalization, ursodeoxycholic acid and silymarin were administered from admission day 5 to day 22 (total: 17 days). On the 10th day of hospitalization, the patient had been receiving ursodeoxycholic acid and silymarin for 5 days.

^b^ Fifteen days after administering ursodeoxycholic acid and silymarin.

^c^ The diagnosis was made a week after discharge, and a combination therapy was initiated immediately after the diagnosis.

The patient was brought to our hospital in 2022 because his liver enzyme levels were discovered to be elevated during health checkups. Upon examination, he was found to be anicteric, and his spleen and liver were discovered to be enlarged on palpation. Abdominal sonography was therefore arranged. The sonography report revealed hepatosplenomegaly with a coarse liver and globular changes in the liver indicative of liver cirrhosis. The patient was referred to our gastrointestinal department for further examination. The results of the liver function tests were as follows: AST level, 105 U/L (normal: ≤34 U/L); alanine aminotransferase (ALT) level, 66 U/L (normal: ≤36 U/L); gamma-glutamyl transpeptidase (GGT) level, 670 U/L (normal: <71 U/L); ALP level, 237 U/L (normal: <122 U/L); total bilirubin level, 1.1 mg/dl (normal: <1.2 mg/dl); direct bilirubin level, 0.5 mg/dl (normal: <0.4 mg/dl); albumin level, 3.7 g/dl (normal: >3.5 g/dl); and ammonia level, 84 µg/dl (normal: <123 µg/dl). The prothrombin time and activated partial thromboplastin time were normal. The patient's white blood cell count and differential count revealed nonspecific findings. The results of virological tests, including those for hepatitis A–C viruses, Epstein–Barr virus, and cytomegalovirus, were negative. His alpha-1 antitrypsin, ceruloplasmin, and ferritin levels were normal. The likelihood of drug-induced liver injury appears to be extremely low after a comprehensive review of the patient's medication history. Given the presence of an unidentified hepatitis with cholestasis complicated by liver cirrhosis, the patient was admitted to our ward for further evaluation.

Magnetic resonance cholangiopancreatography (MRCP) was performed to diagnose possible biliary tract disease. The MRCP revealed masses occupying both lobes of the liver, indicating the presence of severe macronodular cirrhosis. The patient had no bile duct stricture or dilatation and no steatosis. Moreover, the large vessels were patent without any obstruction. Triphasic liver computed tomography accompanied by a thorough study of tumor markers indicated a low possibility of hepatocellular carcinoma. Although the patient had severe liver cirrhosis, he did not display clinical or imaging features of portal hypertension. Upper gastrointestinal endoscopy revealed no esophageal varices. Immunoblot analysis to detect autoimmune liver diseases revealed negative results for circulating autoantibodies, including AMA-M2, anti-soluble liver antigen/liver–pancreas antibodies, anti-liver cytosol type 1 antibodies, anti-liver-kidney microsome type 1 antibodies, anti-smooth muscle antibodies; antinuclear antibodies; and antineutrophil cytoplasmic antibodies. Immunoglobulin (IG) tests revealed slightly elevated IgG levels (1,840 mg/dl, normal: <1,600 mg/dl), with an IgG1 predominance (1,190 mg/dl, normal: <1,011 mg/dl). The patient's IgG4, IgM, and IgA levels were normal. Tests to diagnose Wilson's disease (WD) revealed an increase in the patient's 24-h urinary copper levels, with levels of 57 µg/day and 1,290 µg/day being detected before and after the penicillamine challenge test, respectively (normal: ≤40 µg/day). His serum copper level was also elevated (159 µg/dl, normal: <140 µg/dl). Ophthalmological examination revealed no Kayser–Fleischer rings. Further sequencing of the ATP7B gene revealed normal findings, indicating a low possibility of WD. Brain magnetic resonance imaging revealed increased signal intensity on T1WI over the bilateral globus pallidus, indicative of hepatocerebral degeneration, which may be caused by cholestasis- and liver cirrhosis-related manganese deposition. The serum manganese level was therefore measured and was found to have slightly increased to 2.6 µg/L (normal: <2.4 µg/L).

**Figure 1:**
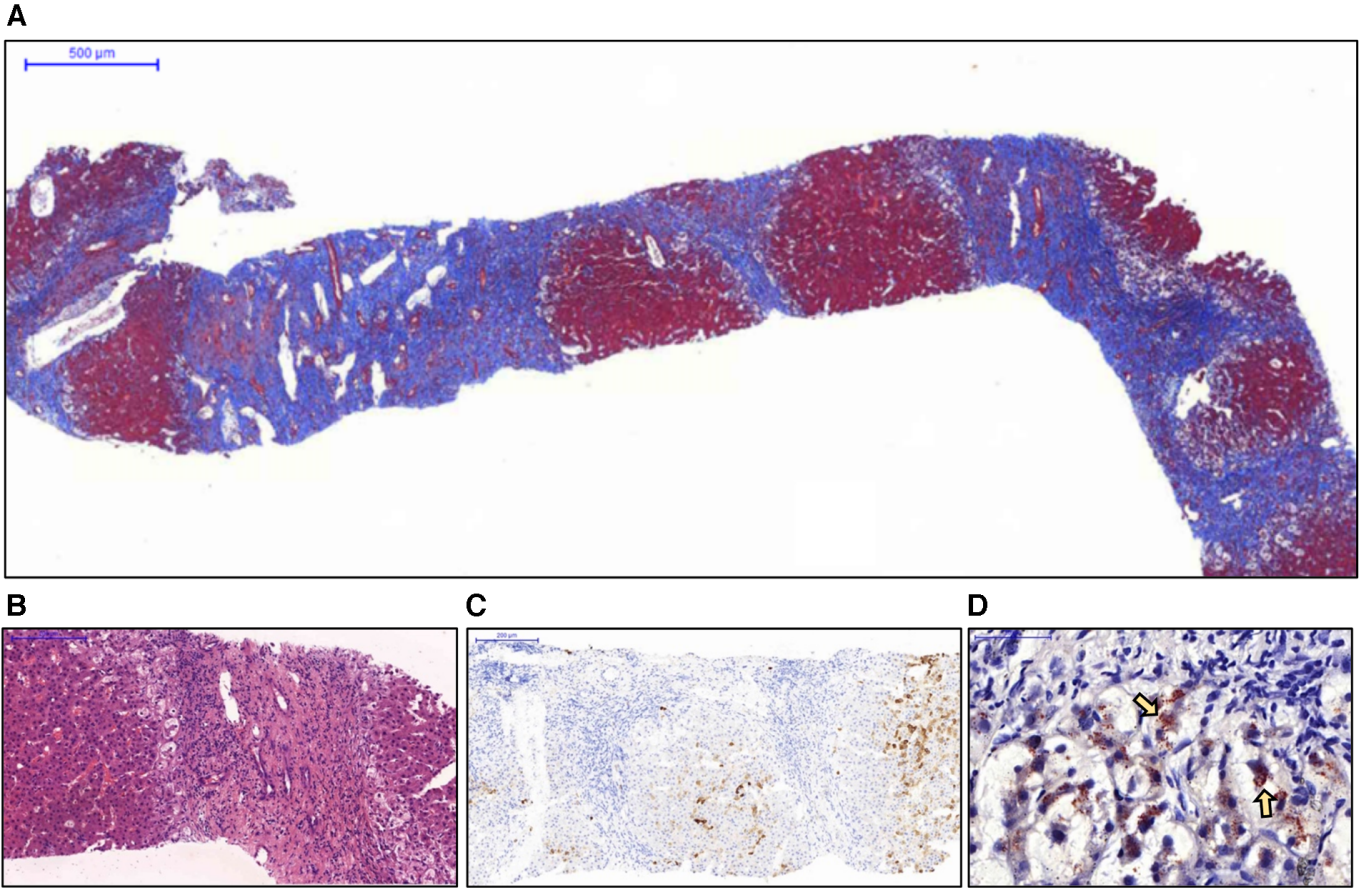
Histopathological examination of the liver biopsy specimen. (**A**) Nodule formation with a thick fibrous septa was noted and considered representative of advanced cirrhosis (Masson's trichrome stain). (**B**) No interlobular bile duct was noted in the enlarged portal tract. Interface inflammation was also noted (hematoxylin and eosin stain). (**C**) Immunohistochemical staining for CK7 revealed a complete absence of bile ducts in the portal tracts. (**D**) The periportal hepatocytes showed marked ballooning changes with abundant copper deposition (arrows) (Rhodanine stain).

Due to the lack of an accurate diagnosis, a liver biopsy was performed. Pathological examination revealed advanced liver cirrhosis (Figure 1A) with a paucity of interlobular bile ducts; this finding was confirmed by the results of immunohistochemical (IHC) staining for CK7 (Figures 1B,C) and a marked increase in copper deposition in the periportal areas (Figure 1D). On the basis of these findings, the patient was considered to have stage IV PBC according to the Ludwig classiﬁcation. Although diffuse and abundant copper deposition was also noted, WD could be ruled out as it is not associated with marked bile duct loss. Interface hepatitis was also noted. Additional IHC staining for CD138 revealed clusters of positive cells (plasma cells) in several portal tracts. Staining for CD20, CD4, and CD8 revealed a moderate-to-marked increase in positive cell infiltration in the portal tracts. These findings were suggestive of significant inflammatory activity and coexisting AIH. However, when progressing to advanced cirrhosis, histopathological findings from different diseases may exhibit similar characteristics. As a result, whole exon sequencing was conducted, which did not identify any pathogenic variant loci associated with potential monogenic hereditary cholestatic liver disorders upon analysis, including the *JAG1* and *NOTCH2* gene for Alagille syndrome, and the *ABCB4* and *TJP2* gene for progressive familial intrahepatic cholestasis type 3 and type 4.

After the diagnosis was made, the patient was administered 13 mg/kg/day ursodeoxycholic acid and 0.5 mg/kg/day prednisolone. Follow-up examinations revealed marked improvements in both cholestasis and liver enzyme levels after 1 month of treatment; these levels have been maintained to date (i.e., one year after the initial treatment). A decrease in the serum copper and manganese levels was also observed (Table 1). Clinically, the patient demonstrated improvement in postural tremor, ataxia and pruritus. Notably, repeated testing of circulating autoantibodies revealed that the patient was positive for AMA-M2 4 months after the treatment. Nevertheless, he was again negative for AMA-M2 6 months after the treatment (Table 1). To determine the possible underlying causes of his multiple congenital abnormalities, array comparative genomic hybridization was performed. The results revealed a 3.89 Mb *de novo* deletions of 14q24.1q24.2, including 22 Online Mendelian Inheritance in Man (OMIM) genes: *RAD51B*, *ZFP36L1*, *ACTN1*, *DCAF5*, *EXD2*, *GALNT16*, *ERH*, *SLC39A9*, *SUSD6*, *SRSF5*, *SLC10A1*, *SMOC1*, *SLC8A3*, *COX16*, *SYNJ2BP*, *ADAM21*, *ADAM20*, *MED6*, *TTC9*, *MAP3K9*, *PCNX1*, and *SIPA1L1*.

## Discussion

We present the case of a 19-year-old male adolescent with a chromosomal defect who was given the diagnosis of stage IV PBC with features of AIH. PBC was diagnosed in accordance with the 2022 Asian Pacific Association for the Study of the Liver (APASL) clinical practice guidance on the basis of the presence of elevated ALP and GGT levels, AMA positivity and typical hepatic histopathological features (1). However, a rigorous criteria for the diagnosis of PBC with AIH features is currently lacking. Though our patient has not yet met the diagnostic requirements for PBC with AIH features according to the 2022 APASL guidance, the pathological findings of severe interface hepatitis, along with the patient's favorable response to treatment with ursodeoxycholic acid and steroids, support the diagnosis.

In addition to the features commonly observed in adults with PBC, our patient had some unusual presentations. First, the onset was early, with elevated AST and ALP levels first observed at age of 3. In the literatures, only 10 pediatric cases of PBC have been reported (Table 2) (5–13), with the mean age at diagnosis being 10.4 years. In these 10 cases, two cases were reported to have had abnormal liver function several years before being diagnosed with PBC: one 16-year-old female who had a slight increase in AST at the age of 11 (6), and another 9-year-old female who had elevated AST and ALT levels at the age of 5 (13). The youngest described case is that of a 3-year-old girl (9) and is the only pediatric case that meets the diagnostic criteria for PBC with AIH features. Second, there appears to be a female predominance in pediatric PBC, with most of the 10 reported cases being girls, and only 2 male cases reported. Third, the AMA status of our patient changed over time. According to the literature, AMA seroconversion occurred in 11%–33% of the initially AMA-negative PBC patients regardless of the testing method used, and these fluctuations did not appear to influence the disease outcomes (14, 15). Fourth, our patient exhibited an increase in serum and urinary copper levels, which may be related to the presence of profound cholestatic diseases with liver cirrhosis. These features were not observed in the other pediatric cases we reviewed, possibly due to a more advanced stage of PBC in our patient, as serum copper levels have been reported to be correlated with the severity of liver cirrhosis (16). Copper and manganese are eliminated through bile under the regulation of liver metabolism. Cholestatic liver disease can therefore affect substances excreted through this pathway, leading to the accumulation of these elements in specific organs. Excessive manganese deposition at the basal ganglia of the central nervous system in patient with liver cirrhosis can cause Parkinson's-like extrapyramidal symptoms. In our patient, postural tremor and ataxia were observed at diagnosis. The serum levels of these elements were reported to decrease after treatment with ursodeoxycholic acid, especially in patients with normalized levels of cholestasis markers after treatment (17). Such an improvement was also noted in our patient, both in laboratory test results (Table 1) and Parkinson's-like symptoms. Copper and manganese levels were positively correlated with the GGT level in our patient. However, there is currently a paucity of literature investigating the relationship between serum copper, manganese, and GGT levels in cirrhotic patients, and further research is needed. The findings in our patient emphasize the importance of regularly monitoring copper and manganese levels in patients with cholestatic or chronic liver diseases, particularly in those with neurological symptoms, significant cholestasis, or advanced-stage disease.

**Table 2:** Summary of case reports of pediatric PBC in the literature.

Reference	Country	Age (yr)	Sex	Diagnosis	Clinical presentation	Family Hx	Autoantibody	Immunoglobulin	Treatment	F/U (yr)	Outcome
Melegh et al. (5)	Hungary	6	F	PBC	Portal hypertension	NR	AMA	NR	NR	5	Died at age 11 (EV rupture)
Dahlan et al. (6)	Canada	16	F	Stage II PBC	Abdominal pain Intractable pruritis Peripheral neuropathy Elevated GGT	Yes^a^	AMA	IgM **826** IgG, IgA NL	UDCA LT at age 21	1	Normal LFTs Symptoms subside after LT
		15	F	Stage II PBC	Abdominal pain Raynaud's phenomenon Sicca symptoms Elevated AST	No	AMA ANA	IgM **696** IgG, IgA NL	UDCA	4	LFTs almost normal
Floreani et al. (7)	Italy	17	F	Stage I PBC	Pruritus Raynaud's phenomenon	No	AMA ANA	IgM, IgG, IgA NL	UDCA	2	Still pruritus sometimes
Aoki et al. (8)	USA	5	M	Stage I PBC	Recurrent infection due to underlying IL2Rα deﬁciency Elevated GGT	NR	AMA ANA	IgG **2,260** IgM, IgA NL	ASCT	5	Complete resolution
Invernizzi et al. (9)	Italy	3	F	Type II AIH with PBC	Jaundice Fatigue Signs of LF	No	AMA ANA LKM	IgG **2,230** IgA **271** IgM NL	Steroid CsA	5	HE complete recovery Normalized LFTs
Kitic et al. (10)	Serbia	12	F	Stage I PBC	Jaundice Fatigue RUQ pain Elevated LFTs	NR	AMA	IgM **229**	UDCA	5	LFTs almost normal 3 mo after treatment
Liberal et al. (11)	Portugal	16	M	Stage I PBC	Elevated LFTs Severe pruritus	No	AMA ANA	IgM **308** IgG, IgA NL	UDCA LT at age 21	NR	NR
Ullah et al. (12)	Pakistan	5	F	PBC	Jaundice Signs of LF	NR	AMA ANA	NR	LT	6 mo	Recover well after LT
Wang et al. (13)	China	9	F	PBC	Pruritis Vomiting Elevated LFTs	No	AMA ANA	IgM, IgG, IgA NL	UDCA	3 mo	LFTs almost normal Symptoms subside

IgG, IgM, and IgA levels are expressed in mg/dl. The results in bold are abnormal.

Yr, years; Mo, months; Hx, history; F/U, follow-up; F, female; M, male; PBC, primary biliary cholangitis; AMA, antimitochondrial antibody; ANA, Anti-nuclear antibody; LKM, Anti-liver-kidney microsomal antibody; NR, not reported; UDCA, ursodeoxycholic acid; CsA, cyclosporine A; RUQ, right upper quadrant; LT, liver transplantation; LFTs, liver function tests; NL, normal; IL2Rα, interleukin-2 receptor alpha chain; ASCT, allogenic stem cell transplantation; GGT, gamma-glutamyl transferase; AST, aspartate aminotransferase; EVs, esophageal varices; LF, liver failure; HE, hepatic encephalopathy.

^a^ The patient's mother had PBC with AIH features and underwent liver transplantation. The patient’s maternal grandmother and great-grandmother died of liver cirrhosis of unknown cause.

Pediatric PBC has rarely been reported in patients with genetic defects. Among the reported pediatric PBC cases, only one 5-year-old boy was noted to have underlying interleukin-2 receptor alpha chain (IL-2Rα) deficiency (8) resulting from a 4-bp deletion over the *IL2RA* gene on chromosome 10. In that patient, complete resolution of PBC and undetectable AMA were noted after allogenic stem cell transplantation. However, that patient was also the only patient in the literature whose genetic test results were reported. Whether other pediatric patients carry genetic abnormalities is unknown. The widespread application of array technologies has provided detailed information on chromosomal abnormalities. However, in some chromosomal regions, such as the interstitial deletions on chromosome band 14q24.1q24.2 in our patient, no well-characterized aberrations have been discovered. To the best of our knowledge, a patient with 14q24.1q24.2 deletions has never been reported; however, two case reports have been published on four unrelated pediatric patients with *de novo* 14q24.1q24.3 deletions (18, 19). Our case shared similar facial dysmorphism and organ abnormalities with them; however, none of the reported cases involved liver diseases. PBC susceptibility–related human leukocyte antigen (HLA) and non-HLA loci have been discovered through genomewide association studies (20). Mells et al. (21) reported that the *RAD51L1* gene located on chromosome 14q24 a confirmed risk locus for PBC. Although the *RAD51L1* gene was not precisely located at the deleted site in our patient, the 22 OMIM genes affected in our patient may include one or more risk loci for PBC.

Younger age at presentation is considered a poor prognostic factor in PBC (22). Among the pediatric cases we reviewed, one 6-year-old patient died of complications from liver disease five years after diagnosis, two 16-year-old patients underwent liver transplantation five years after treatment with ursodeoxycholic acid, and one 5-year-old patient soon developed hepatic encephalopathy and subsequently underwent liver transplantation (5, 6, 11, 12). In addition to the age at onset, our patient has other risk factors that contribute to a poorer long-term prognosis (1), including baseline cirrhosis, ductopenia observed in histological analysis, and the presence of PBC with AIH features, which indicates a significant likelihood of eventual liver transplantation. Furthermore, the risk of hepatocellular carcinoma is significantly higher in PBC patients, as demonstrated by a systematic review showing a pooled relative risk of 18.80 compared to the general population, especially in males or those with cirrhosis (23). Therefore, long-term follow-up may be warranted.

## Conclusion

The true incidence and natural history of PBC and PBC with AIH features in pediatric patients remain unknown. The present case can increase the awareness of early-onset PBC and its possible correlation with chromosomal defects.

## Data Availability

The original contributions presented in the study are included in the article/Supplementary Material, further inquiries can be directed to the corresponding author.
